# The effect of wheat prebiotics on the gut bacterial population and iron status of iron deficient broiler chickens

**DOI:** 10.1186/1475-2891-13-58

**Published:** 2014-06-13

**Authors:** Elad Tako, Raymond P Glahn, Marija Knez, James CR Stangoulis

**Affiliations:** 1 USDA/ARS, Robert W. Holley Centre for Agriculture and Health, Cornell University, Ithaca, NY 14853, USA; 2 School of Biological Sciences, Flinders University, GPO Box 2100, Adelaide, SA 5001, Australia

**Keywords:** Prebiotics, Arabinoxylan, Probiotics, Iron bioavailability, Chicken, Caco-2 cells

## Abstract

**Background:**

Currently, there is a lot of interest in improving gut health, and consequently increasing Fe absorption, by managing the colonic microbial population. This is traditionally done by the consumption of probiotics, live microbial food supplements. However, an alternative, and often very effective approach, is the consumption of food ingredients known as prebiotics. Fructans and arabinoxylans are naturally occurring non-digestible oligosaccharides in wheat that exhibit prebiotic properties and may enhance intestinal iron (Fe) absorption. The aim of this study was to assess the effect of prebiotics from wheat on Fe bioavailability *in vitro* (Caco-2 cells) and *in vivo* (broiler chickens, Gallus gallus).

**Methods:**

In the current study, the effect of intra-amniotic administration of wheat samples extracts at 17 d of embryonic incubation on the Fe status and possible changes in the bacterial population in intestinal content of broiler hatchlings were investigated. A group of 144 eggs were injected with the specified solution (1 ml per egg) into the amniotic fluid. Immediately after hatch (21 d) and from each treatment group, 10 chicks were euthanized and their small intestine, liver and cecum were removed for relative mRNA abundance of intestinal Fe related transporters, relative liver ferritin amounts and bacterial analysis of cecal content, respectively.

**Results:**

The *in vivo* results are in agreement with the *in vitro* observations, showing no differences in the hatchling Fe status between the treatment groups, as Fe bioavailability was not increased *in vitro* and no significant differences were measured in the intestinal expression of DMT1, Ferroportin and DcytB *in vivo*. However, there was significant variation in relative amounts of bifidobacteria and lactobacilli in the intestinal content between the treatments groups, with generally more bifidobacteria being produced with increased prebiotic content.

**Conclusions:**

In this study we showed that prebiotics naturally found in wheat grains/bread products significantly increased intestinal beneficial bacterial population in Fe deficient broiler chickens. With this short-term feeding trial we were not able to show differences in the Fe-status of broilers. Nevertheless, the increase in relative amounts of bifidobacteria and lactobacilli in the presence of wheat prebiotics is an important finding as these bacterial populations may affect Fe bioavailability in long-term studies.

## Introduction

Micronutrient malnutrition, often called ‘hidden hunger’, is a serious health problem worldwide. The most prevalent micronutrient deficiency is iron (Fe) deficiency, affecting about 40% of the world’s population, particularly women and children in developing countries [1,2]. Cereal crops are an important source of minerals and other nutrients for humans. Wheat is currently the primary staple food for nearly one-third of the world’s population, providing >50% of the total daily energy intake [3]. Wheat grains contain significant amounts of dietary fiber and phytate.

It is believed that diets high in phytate contribute to Fe deficiency [4,5]. In contrast, some naturally occurring non-digestible oligosaccharides in wheat, known as prebiotics, have been suggested to have an enhancing effect on Fe absorption [6,7].

Prebiotics have the ability to support the growth of probiotics [8,9]. The fermentation of prebiotics by colonic bacteria gives rise to production of unbranched SCFA such as acetic, propionic, butyric, and lactic acids, thereby lowering intestinal pH [10,11], inhibiting the growth of potentially harmful bacteria [11] and improving mineral absorption [12-15]. Next to inulin and fructo-oligosaccharides (FOS), the two most extensively studied prebiotics, arabinoxylans (AXs) are also considered as a potential new class of prebiotic components [16]. AXs are present mainly in the bran portion, for example in wheat bran (6.7%). However, the thin aleurone layer surrounding wheat endosperm contains 60-70% AX [17]. Overall, they constitute 60-69% of non-starch polysacharides in wheat bran [18] and 88% in wheat endosperm [19].

Inulin and FOS have been shown several times to selectively stimulate bifidobacteria [20-22,7]. Regarding the absorption of Fe, it has recently been shown that intra-amniotic administration and dietary inulin improved the Fe status of Fe deficient broilers [9]. mRNA abundance of DMT1 and ferroportin in addition to liver ferritin were higher in the inulin group compared to control (p < 0.05) [9]. Similarly, Yasuda et al. [15] demonstrated improvement in hemoglobin repletion efficiency in young anemic pigs fed corn soybean meal supplemented with 4% dietary inulin. Recently, Freitas et al. [23] showed the benefit of inulin and oligofructose prebiotics on the regeneration of haemoglobinic mass and increased intestinal Fe absorption in anaemic rats. However, to date, the comparison of the effect of prebiotics (with different fructan and arabinoxylan content) both from raw and processed wheat on Fe absorption has not been investigated.

Therefore, the primary aim of this study was to assess the effect of prebiotics from wheat on short-term Fe bioavailability *in-vitro* (in Caco-2 cells) and *in-vivo* (broiler chickens). Within this aim there were several objectives: (A) to compare the effect of wheat treatments with varied arabinoxylan and fructan content on Fe availability and (B) to evaluate the differences in the efficacy of prebiotics from processed and unprocessed wheat grain flour. Finally, in view of the fact that people do not consume raw cereal grains and that processing and baking are common practices worldwide, our last objective was (C) to examine the effect of prebiotics from baked wheat products (breads baked for 45 min and 5 min) on Fe status and gut microbiata of iron deficient broilers hatchlings.

## Materials and methods

### Wheat and bread samples

The four samples of double haploid lines included in this study were from the Berkut × Krichauff doubled haploid (DH) population, previously used to map quantitative trait loci (QTL) for high AX in this population [24]. The population was grown in 2009 at Roseworthy in South Australia, at an adequate-rainfall site. The lines were chosen based on their fructan and arabinoxylan content (the lines with the highest and the lowest fructan and arabinoxylan amounts were included). These samples were compared with commercially bought white and wholemeal flour mixes and their baked products (Laucke Flour Mills; http://www.laucke. com.au).

### Bread and chapatti sample preparation

Bread loaves (1 kg, standard size) were baked using an automatic bread maker Breville BBM100 (Sydney, Australia). An identical baking program was established for the preparation of all breads.

The endpoint of baking was set as “Medium Crust.” The total baking time was 3 h and 5 min, including 25 min kneading, 1 h and 40 min fermentation and 1 h baking at 155°C.

The average amount of the flour mix used for preparation of a loaf of bread was 600 g.

Weight of other ingredients (yeast and water) was 420 g for white and 550 g for wholemeal bread. Once the baking was completed bread loaves were removed from the oven, left to cool on a rack and subsequently frozen to −25°C.

Chapatti breads were prepared using 120 g of the flour mix and 80 ml of water. Dough was kneaded by hand for 20 min than left to rest for a further 30 min (room temperature) before cooking. The dough was shaped into a flat 6-inch disc and cooked on a hot skillet (Sunbeam Classic Skillet SK 4200P 25 cm; 210°C) for 1–2 min until brown spots formed on both sides of the dough. Cooked chapatti was left to cool to room temperature and put at −25°C freezer.

The samples (both yeast leavened and chapatti breads) were freeze-dried for 48 h (Dynavac freeze drying unit) to remove all moisture before grinding (Retch 2M1000b; Retch, Ochten, The Netherlands). All milled samples were stored in plastic containers at room temperature until analysis.

### Sugar, phytate and micronutrient analyses

Sugar and phytate analyses were performed using High Performance Liquid Chromatography (HPLC) on a Dionex ICS-3000 unit as described by Huynh et al. [25] for fructans, Nguyen et al. [24] for AXs and by Lehrfeld et al. [5] for phytate. The micronutrient concentration of samples was determined by inductively coupled plasma optical emission spectrometry (ICP-OES) [26] using an ARL 35B ICP analyser.

### Extraction of the wheat samples

Procedure was conducted as previously described by Vidanarachchi et al. [27]. Briefly, the wheat exudate was dissolved in distilled water (50 g/L) and the solution was filtered through a 600 μm screen to remove particulate matter and then centrifuged at 12,000 g for 10 min at 4°C. The supernatant was collected and dialysed (MWCO 12–14 kDa, Medicell International Ltd., London, UK) exhaustively against distilled water (48 h). The dialysate was collected and then lyophilized to yield a fine off-white powder (wheat extract). In addition, in order to avoid embryonic dehydration, the samples were diluted prior to *in-ovo* injections (solution concentration was not higher than 320 OSM).

### *In vitro* iron bioavailability assessment

An *in vitro* digestion/Caco-2 cell culture model [28] was used to assess *in vitro* Fe bioavailability. With this method, bread/flour samples (0.5 g) were subjected to simulated gastric and intestinal digestion.

Briefly, the intestinal digestion is carried out in cylindrical inserts closed on the bottom by a semipermeable membrane and placed in wells containing Caco-2 cell monolayers bathed in culture medium. The upper chamber was formed by fitting the bottom of Transwell insert ring (Corning) with a 15000 Da molecular weight cut off (MWCO) membrane (Spectra/Por 2.1, Spectrum Medical, Gardena, CA). The dialysis membrane was held in place using a silicone ring (Web Seal, Rochester, NY).

Iron uptake by the cell monolayers was assessed by measuring ferritin concentrations in the cells. Three replicates of each Fe bioavailability measurement were performed, each utilizing a separate sample of the wheat extract.

In terms of materials for the study, Caco-2 cells were obtained from the American Type Culture Collection (Rockville, MD) at passage 17 and used in experiments at passage 29. Cells were seeded at densities of 50,000 cells/cm2 in collagen-treated 6 well plates (Costar Corp., Cambridge, MA). The integrity of the monolayer was verified by optical microscopy. The cells were cultured at 37°C in an incubator with 5% CO2 and 95% air atmosphere at constant humidity, and the medium was changed every 48 h.

The cells were maintained in Dulbecco’s modified Eagle medium plus, 25 mmol/L HEPES, and 10% fetal bovine serum. 48 h prior the experiment, the growth medium was removed from culture wells, the cell layer was washed, and the growth medium was replaced with minimum essential media (MEM) at pH 7.0.

The MEM was supplemented with 10 mmol/L PIPES, 1% antibiotic/antimycotic solution, 4 mg/L hydrocortisone, 5 mg/L insulin, 5 μg/L selenium, 34 μg/L triiodothyronine, and 20 μg/L epidermal growth factor. This enriched MEM contained less than 80 μg Fe/L.

All ingredients and supplements for cell culture media were obtained from Gibco (Rockville, MD). The cells were used in the Fe uptake experiment at 13 days post seeding. In these conditions, the amount of cell protein measured in each well was highly consistent between wells. In experiment day, 1.5 mL of the digested sample was added to the insert’s upper chamber and incubated for 2 h. Then, inserts were removed and 1 mL of MEM was added. Cell cultures were incubated for 22 h at 37°C.

### Harvesting of Caco-2 cells for ferritin analysis

The protocols used in the ferritin and total protein contents analyses of Caco-2 cells were similar to those previously described [8,29,9,28,30].

Briefly, growth medium was first removed from the culture well by aspiration and the cells were washed twice with a solution containing 140 mmol/L NaCl, 5 mmol/L KCl, and 10 mmol/L PIPES at pH 7.0. The cells were harvested by adding an aliquot of deionized water and placing them in a sonicator (Lab-Line instruments, Melrose Park, IL). The ferritin and total protein concentrations were determined on an aliquot of the harvested cell suspension with a one-stage sandwich immunoradiometric assay (FER-IRON II Ferritin assay, Ramco laboratories, Houston, TX) and a colorimetric assay (Bio-Rad DC Protein assay, Bio-Rad, Hercules, CA), respectively.

Caco-2 cells synthesize ferritin in response to increases in intracellular iron concentration. Therefore, we used the ratio of ferritin/total protein (expressed as ng ferritin/mg protein) as an index of the cellular Fe uptake. All glassware used in the sample preparation and analyses was acid washed.

### *In-vivo* (intra amniotic administration procedure) bioavailability analysis

#### Birds, diets, and study design

One hundred and sixty Cornish-cross fertile broiler eggs were obtained from a commercial hatchery (Moyer’s chicks, Quakertown, PA), from a maternal flock 35 weeks in lay. The eggs were incubated under optimal conditions at the Cornell University Animal Science poultry farm incubator.

### Intra-amniotic administration

The intra amniotic administration procedure was previously described [9,31]. Briefly, at 17 days of embryonic incubation, eggs containing viable embryos (n = 150) were weighed and divided into 13 groups (n = 12), each with an average egg weight of 49 ± 1.15 g.

All treatment groups were assigned eggs of similar weight-frequency distribution.

A group of 144 eggs was then injected with the specified solution (1 ml per egg) with a 21-gauge needle into the amniotic fluid, which was identified by candling [31]. The intra-amniotic treatment solution included the following: ‘Control 1’: non injected eggs; ‘Control 2’: inulin solution (4%-inulin/ddH2O); ‘Control 3’: arabinose solution (4%-arabinose/ddH2O) and 10 samples (extracted in ddH2O).

‘Control 1’ (n = 12) was non injected group that paralleled routine procedures in commercial hatcheries. After all the eggs were injected, the injection holes were sealed with cellophane tape and the eggs placed in hatching baskets such that each treatment was equally represented at each incubator location.

Hatchability was similar in all treatment groups and was approximately 90%. Immediately after hatch (21 d) and from each treatment group, 10 chicks were euthanized by carbon dioxide exposure. The digestive tracts and livers were quickly removed from the carcass and separated into various sections for tissue (small intestine and liver ~ 1-2 cm; ~2-3gr, respectively) and cecum.

Samples were collected for relative mRNA abundance of intestinal Fe related transporters, relative liver ferritin amounts and bacterial analysis of cecal content, respectively. The samples were immediately frozen in liquid nitrogen, and then stored in a −80°C freezer until analysis. All animal protocols were approved by the Cornell University Institutional Animal Care and Use Committee.

### Blood analysis and hemoglobin (Hb) measurements

Blood samples were collected weekly from the wing vein (n = 10, ~50 μL) using micro-hematocrit heparinized capillary tubes (Fisher, Pittsburgh, PA). Samples were collected immediately after hatch. Blood Hb concentrations were determined spectrophotometrically using the cyanmethemoglobin method (H7506-STD, Pointe Scientific Inc. Canton, MI) following the kit manufacturer’s instructions.

### Isolation of total RNA

Total RNA was extracted from 30 mg of the duodenal tissue (tissue was harvested from the proximal duodenum, n = 10) using QiagenRNeasy Mini Kit (RNeasy Mini Kit, QiagenInc.,Valencia, CA) according to the manufacturer’s protocol. Briefly, tissues were disrupted and homogenized with a rotor-stator homogenizer in buffer RLT®, containing β-mercaptoethanol. The tissue lysate was centrifuged for 3 minutes at 8,000 g in a micro centrifuge. An aliquot of the supernatant was transferred to another tube, combined with 1 volume of 70% ethanol and mixed immediately.

Each sample (700 μL) was applied to an RNeasy mini column, centrifuged for 15 s at 8000 g, and the flow through material was discarded. Next, the RN easy columns were transferred to new 2-mL collection tubes, and 500 μL of buffer RPE® was pipetted onto the RNeasy column followed by centrifugation for 15 s at 8000 g. An additional 500 μL of buffer RPE were pipetted onto the RNeasy column and centrifuged for 2 min at 8000 g.

Total RNA was eluted in 50 μL of RNase free water. All steps were carried out under RNase free conditions. RNA was quantified by absorbance at A 260/280. Integrity of the 28S and 18S ribosomal RNAs was verified by 1.5% agarose gel electrophoresis followed by ethidium bromide staining.

DNA contamination was removed using TURBO DNase treatment and removal kit from AMBION (Austin, TX, USA).

### DMT1, DcytB and Ferroprtin gene expression analysis

As previously described [8,29,32,9], briefly, PCR was carried out with primers chosen from the fragment of the chicken (Gallus gallus) duodenal DMT1 gene (GeneBank database; GI 206597489) (forward: 5’-AGC CGT TCA CCA CTT ATT TCG-3’; reverse: 5’-GGT CCA AAT AGG CGA TGC TC-3’), DcytB gene (GI 20380692) (forward: 5’-GGC CGT GTT TGA GAA CCA CAA TGT T-3’; reverse: 5’-CGT TTG CAA TCA CGT TTC CAA AGA T-3’) and Ferroportin gene (GI 61098365) (forward: 5’-GAT GCA TTC TGA ACA ACC AAG GA’; reverse: 5’-GGA GAC TGG GTG GAC AAG AAC TC-3’). Ribosomal 18S was used to normalize the results (GI 7262899) (forward: 5’- CGA TGC TCT TAA CTG AGT-3’; reverse: 5’-CAG CTT TGC AAC CAT ACT C-3’).

Determination of the linear phase of the PCR amplification was performed (Access RT-PCR system, Promega, Madison, WI) with pooled aliquots removed at 15, 20, 25, 30, 35, 40, 45, 50, and 55 cycles.

Amplification of the chicken duodenal DMT1, DcytB and Ferroportin genes were performed for 32, 40 and 30 cycles respectively, which consisted of denaturation (95°C, 30 s), annealing (48°C, 1 min),

and extension (72°C, 1 min); ribosomal 18S was amplified at 32 cycles under identical conditions in a different tube. All PCR products were separated by electrophoresis on 2% agarose gel, stained with ethidium bromide, and quantified using the Quantity One 1-D analysis software (Bio-Rad, Hercules, CA).

### Ferritin and Fe in the liver

Liver samples were treated as described by Mete et al. [33]. Briefly, 1 g of sample was diluted into 1 mL of 50 mM Hepes buffer, pH 7.4, and homogenized on ice at 5000 g and for 2 min. One mL of each homogenate was subjected to heat treatment for 10 min at 75°C to aid isolation of ferritin since other proteins are not stable at that temperature [33,34,32,9]. After heat treatment the samples were immediately cooled down on ice for 30 min.

Thereafter, samples were centrifuged at 13000 g for 30 min at 4°C until a clear supernatant was obtained and the pellet containing most of the insoluble denaturated proteins was discarded. All tests were conducted in duplicates for each animal (n = 6).

### Electrophoresis, staining and measurement of gels

Native polyacrylamide gel electrophoresis was conducted using a 6% separating gel and a 5% stacking gel. Samples were run at a constant voltage of 100 V. After electrophoresis, the gels were treated with either of the two stains: Coomasie blue G-250 stain, specific for proteins, or potassium ferricyanide (K3Fe(CN)6) stain, specific for iron.

The corresponding band found in the protein and iron stained gel was considered to be ferritin [32,35,34]. The gels were scanned with Bio-Rad densitometer. Measurements of the bands were conducted using the Quantity-One 1-D analysis program (Bio-Rad, Hercules, CA). The local background was subtracted from each sample.

Horse spleen ferritin (Sigma Aldrich Co., St. Louis, MO) was used as a standard for calibrating ferritin protein and iron concentrations of the samples. Dilutions of the horse spleen ferritin were made and treated similarly to the liver supernatant samples in order to create a reference line for both protein and iron-stained gels. Iron levels were determined using the same calibration since approximately 20% of the weight of horse spleen ferritin is iron.

Saturation levels of ferritin with iron were calculated as the percentage of the iron present in the protein to the maximum amount of iron atoms (4500 iron atoms/ferritin molecule) ferritin can incorporate [32,35,34].

### Collection of microbial samples and DNA isolation

The cecum was removed and treated as described previously (Zhu et al., 2002; Tako et al., 2008; Tako and Glahn, 2012). The contents of the cecum were squeezed out into a sterile 50-mL tube containing 9 mL of sterile PBS and homogenized by vortexing with glass beads (3-mm diameter) for 3 min.

Debris was removed by centrifugation at 700 × g for 1 min, and the supernatant was collected and centrifuged at 12,000 × g for 5 min. The pellet was washed twice with PBS and stored at −20°C until DNA extraction. For DNA purification, the pellet was resuspended in 50 mM EDTA and treated with lysozyme (Sigma Aldrich Co., St. Louis, MO; final concentration of 10 mg/mL) for 45 min at 37°C. The bacterial genomic DNA was isolated using a Wizard Genomic DNA purification kit (Promega Corp., Madison, WI). The DNA concentration was determined spectrophotometrically.

### Primer design and PCR amplification of bacterial 16S rDNA

Primers for Lactobacillus (forward: 5’- CAT CCA GTG CAA ACC TAA GAG-3’; reverse: 5’- GAT CCG GTG CAA ACC TAA GAG-3’) and Bifidobacterium (forward: 5’- GGG TGG TAA TGC CGG ATG-3’; reverse: 5’- CCA CCG TTA CAC CGG GAA-3’) were designed according to previously published data [36-38,9,35]. Universal primers identifying all known bacteria were designed using the invariant region in the 16 s rDNA of the bacteria (forward: 5’- CGT GCC AGC CGC GGT AAT ACG -3’; reverse: 5’- CGT GCC AGC CGC GGT AAT ACG-3’). The universal primer set was used for determining the total microflora population. For PCR amplification of the bacterial targets from cecal contents, 5 μL of DNA extract was added to 45 μL of PCR mixture containing 27.5 μL of nuclease-free water, 5 μL of each primer (10 μg/mL), 1.5 μL of nucleotide (dNTP) mix, 5 μL of PCR buffer, and 1 μL of Taq polymerase (Go-Taq, Promega). The PCR thermal conditions were as follows: 1 cycle of 94°C for 3 min, 35 cycles of 94°C for 30 s, 60°C for 1 min, 68°C for 2 min, and finally 1 cycle of 68°C for 7 min. The PCR reaction was run with different numbers of cycles (25, 30, 35, 40, 45, or 50) for each primer set, and 35 cycles was in the center of the exponential increase in PCR products.

The PCR products were separated by electrophoresis on 2% agarose gel, stained with ethidium bromide, and quantified using a Gel-Pro analyzer version 3.0 (Media Cybernetics, Bethesda, MD). To evaluate the relative proportion of each examined bacteria, all products were expressed relative to the content of the universal primer product and proportions of each bacterial group are presented, where the total of the examined bacteria was set at 100%.

### Statistical analysis

Results were analyzed by one way ANOVA using the SPSS statistical software (SPSS 17.0; SPSS Inc, Chicago, IL). Differences between treatments were compared by Tukey’s test and significance level of p < 0.05 was used for all comparisons. Values in the text are means ± SD.

## Results and discussion

### The content of prebiotics and phytate and the concentration of micronutrients in wheat samples

Among the DH lines there were samples with similar levels of phytate and the content of fructans plus arabinoxylans varying from 7.7% to 10.6% (Table 1).

**Table 1:** The content of prebiotics (arabinoxylans and fructans) and phytate, and the concentration of micronutrients (zinc, iron, phosphorous) in various flour and bread samples

**Sample**	**AX (%)**	**Fructan (%)**	**Phytate (%)**	**Zn (mg/kg)**	**Fe (mg/kg)**	**P (mg/kg)**
DH line number 7	7.9 ± 0.01	2.7 ± 0.03	1.49 ± 0.01	28	41	4200
DH line number 121	5.8 ± 0.04	1.9 ± 0.01	1.41 ± 0.03	29	47	4300
DH line number 114	5.8 ± 0.10	3.2 ± 0.05	1.29 ± 0.04	25	39	3800
DH line number 150	7.2 ± 0.03	0.9 ± 0.03	1.15 ± 0.01	25	36	3800
Crusty white bread mix flour	1.7 ± 0.11	1.5 ± 0.05	0.19 ± 0.03	6.9	11	1150
White yeast leavened bread	1.6 ± 0.06	0.6 ± 0.38	0.01 ± 0.02	8.2	12	1330
White chapatti bread	1.5 ± 0.03	1.5 ± 0.14	0.15 ± 0.04	14	19	1700
Wholemeal bread mix flour	3.8 ± 0.03	2.0 ± 0.20	0.72 ± 0.01	18	33	2600
Wholemeal yeast leavened bread	3.9 ± 0.06	1.0 ± 0.38	0.37 ± 0.03	20	37	3000
Wholemeal chapatti bread	3.5 ± 0.26	1.9 ± 0.34	0.64 ± 0.03	19	34	2800

Values are means +/− SD (n = 4). AX = arabinoxylan.

To examine the effect of prebiotics from processed wheat (and compare them with prebiotic effects of raw material) two commercially bought flour samples were included in this study (Laucke's white and wholemeal flour). These samples contained lower amounts of fructans and arabinoxylans (3.2% and 5.8% fructan plus arabinoxylan), but also much lower levels of phytates. In order to get a better insight into the prebiotic efficacy of baked products we tested the samples of leavened white and wholemeal yeast bread, and chapatti bread.

Yeast leavened breads are most frequently consumed breads in Western countries, while chapatti is the most common type of unleavened wheat bread consumed in Asia. In Pakistan, for example, more than 70% of the total wheat produced is used to make chapattis [39]. Baked samples of yeast leavened breads contained lower levels of phytate (phytate is degraded during bread baking processes, from 50% to 90%), and lower levels of prebiotics (30%, fructan reduction, no changes in the content of arabinoxylans), when compared with their corresponding flour samples (Table 1).

There were similar amounts of arabinoxylan, fructan and phytate in the flour and their analogous chapatti breads. The concentrations of micronutrients were lowest in white flour samples and highest in DH line samples.

### *In vitro* bioavailability results

**Figure 1:**
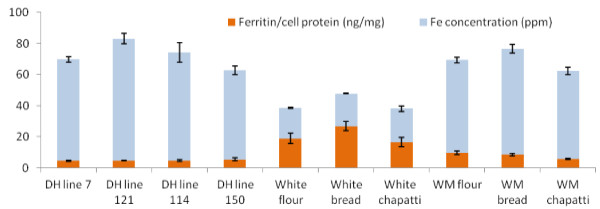
**Summary of *in vitro *bioavailability results.** Red columns - Ferritin concentrations in Caco-2 cells exposed to samples (ng of ferritin/mg of protein). Values are means +/− SD (n = 6), P < 0.05. Blue columns - Sample Fe concentration (means +/− SD (n = 3), P < 0.05, ppm). WM = wholemeal.

The quality controls of *in vitro* study (ie. cell baseline, FeCl3, FeCl3 + AA) were all within the expected and relative ranges which indicate a valid and functional run of the bioassay. As predicted, the results showed improved Fe bioavailability in the presence of white and wholemeal flour and bread samples (Figure 1). The inhibitory effect of phytate on Fe bioavailability is well known [40,41]. Therefore, the lower amounts of phytate in white and wholemeal samples (compared to DH lines) are most likely responsible for observed enhanced Fe bioavailability.

The results of this study confirm previous research [42,30] that reduction of phytate increases iron bioavailability in Caco-2 cells and one would expect enhanced availability if tested in the animal gut. Wheat grains contain endogenous phytase, an enzyme that can release covalently bound phosphate groups from the inositol ring, thus reducing the anti-nutritional effect of phytic acid [43].

These enzymes act by hydrolysing phytic acid into lower myo-inositol phosphate esters with a lower capacity to bind minerals [44]. The fermentation process provides the optimal conditions for phytase activity.

During conversion of flour into bread, phytate content decreases as a consequence of the activity of native phytase. However, as shown in this study the phytate is not reduced to an extent as to greatly improve Fe bioavailability in whole-wheat products. Different factors determine the reduction of phytate content during baking processes such as phytase activity, dough pH, presence of calcium salts and the degree of flour extraction [45].

The essential factor that determines the bioavailability of a compound is the release and solubility of the compound from the food matrix [42]. Even so, relative to controls, bioavailability values of all wheat samples examined in this study were low. Based on previous research, we can assume that such low values (ie. close to baseline, or below) are due to the presence of inhibitors, in this case phytate. However, it should be noted that Fe availability of wheat products is not affected entirely by phytic acid concentration and that other components of wheat grains and bakery products may also have an effect on iron availability; i.e. proteins, calcium [42].

### *In vivo* bioavailability results (DMT1, DcytB, ferroportin, liver ferritin, Hb)

There were no significant differences in Fe bioavailability between treatment diets for hemoglobin (Table 2).

**Table 2:** Blood hemoglobin (Hb) concentration (g/dL), liver ferritin protein amounts, and duodenal mRNA abundance of DMT1, Dcytb and ferroportin in broiler chickens fed with different flour and bread samples

**Sample**	**Blood Hb**	**Liver ferritin**	**Dcytb**	**DMT1**	**Ferroportin**
DH line number 7	10.22 ± 1.40	0.56 ± 0.02	0.65 ± 0.04	0.75 ± 0.05	0.69 ± 0.02
DH line number 121	11.31 ± 1.40	0.56 ± 0.02	0.65 ± 0.04	0.75 ± 0.04	0.68 ± 0.04
DH line number 114	10.50 ± 1.65	0.55 ± 0.04	0.65 ± 0.03	0.75 ± 0.04	0.68 ± 0.03
DH line number 150	10.91 ± 2.05	0.56 ± 0.03	0.65 ± 0.02	0.76 ± 0.04	0.69 ± 0.02
Crusty white bread mix flour	10.64 ± 1.79	0.55 ± 0.03	0.65 ± 0.03	0.76 ± 0.03	0.69 ± 0.03
White yeast leavened bread	11.13 ± 1.50	0.55 ± 0.03	0.65 ± 0.04	0.76 ± 0.04	0.69 ± 0.03
White chapatti bread	11.59 ± 0.88	0.55 ± 0.04	0.66 ± 0.05	0.76 ± 0.04	0.69 ± 0.04
Wholemeal bread mix flour	11.32 ± 1.39	0.55 ± 0.03	0.66 ± 0.03	0.75 ± 0.03	0.69 ± 0.03
Wholemeal yeast leavened bread	11.59 ± 1.36	0.56 ± 0.02	0.65 ± 0.04	0.76 ± 0.03	0.69 ± 0.03
Wholemeal chapatti bread	10.61 ± 0.89	0.56 ± 0.04	0.65 ± 0.04	0.76 ± 0.02	0.69 ± 0.02

Changes in mRNA abundance are expressed relative to the expression of 18S rRNA in arbitrary units (AU). Values are means +/− SD (n = 10).

Similarly, no significant variations were observed in the intestinal expression of DMT1, Ferroportin (major intestinal Fe transporters) and DcytB (Fe reductase). The relative liver ferritin amounts between the treatment groups were also not different. One possible explanation is the amount of material that was administrated *in-ovo*, as we were limited by the osmolality of the injected solution (not more than 320OSM, as embryonic dehydration will occur).

Another reason could be the duration of the study; it was a short term (one time administration) study, which may not be long enough time to observe the efficacy of these particular treatments on Fe bioavailability measures. Similar to previous studies, the poultry [29,9] and Caco-2 cell [28,30] models are sensitive and responsive to bioavailable Fe.

In conclusion, the *in vivo* results are in agreement with the *in vitro* observations showing no differences in the hatchling Fe status between the treatment groups.

### Bacterial populations in the intestinal content of chickens

**Figure 2:**
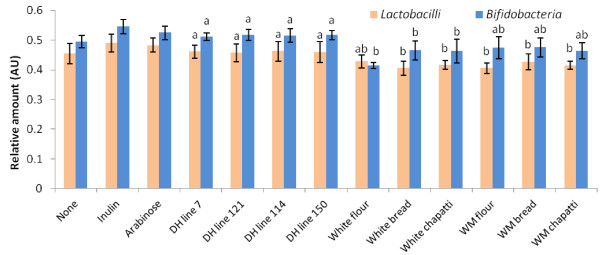
**The relative amounts of Lactobacilli and Bifidobacteria generas in intestinal contents of chickens expressed in arbitrary units (AU).** Values are means +/− SD (n = 10). a, b Mean values within each bacterial species tested with unlikesuperscript letters are significantly different (p < 0.05).

Overall, there was a significant increase in the relative amounts of bifidobacteria and lactobacilli in intestinal content between the treatments groups (Figure 2). Considerably more bifidobacteria has been produced in the presence of most samples (exception is white flour sample where there were no statistically significant differences in the production of bifidobacteria and lactobacilli).

Making individual comparisons, there were no statistically significant differences in both lactobacilli and bifidobacteria production between DH line treatments. As expected, due to the higher amounts of prebiotics present (difference in prebiotic content up to 7.4%) there were significantly higher production of bacteria in the presence of DH line treatments compared to the treatments with commercially produced white and wholemeal flour samples (p < 0.01).

There were statistically significant difference in expansion of bacteria between the wholemeal and white flour (wholemeal more bifidobacteria, white more lactobacillus) (p = 0.02; p = 0.04, respectively).

Additionally, there were no discrepancies in lactobacillus production among the treatments of white and wholemeal flour and their corresponding baked products.

In the presence of white flour baked products, more bifidobacteria was produced in the white flour treatment (p < 0.003); while there were no changes in production of bifidobacteria between the wholemeal flour and corresponding baked products. Finally, there were no variations in bacterial production between the white and wholemeal breads.

In conclusion, prebiotic segments of wheat enhanced the growth of colon microflora, with generally more bifidobacteria being formed. This is an important finding as these bacterial populations may affect Fe bioavailability and improve gut health.

Our findings are concurring with previously published papers that demonstrate an increase in bifidobacteria population in the ceca in the presence of inulin [29,46,47]. Some recently completed human trials clearly show the association between the production of gut bacteria and Fe absorption.

In Fe-deficient young women in South India lower levels of lactobacilli were measured which suggest that bacterial flora have an important role in the Fe absorption process [48]. In addition, it has been demonstrated that bifidobacteria have a nutritional advantage compared with other intestinal microorganisms due to their β-1,2-glycosidase activity [49,7].

As mentioned earlier, one of the possible reasons for increased mineral bioavailability in the presence of prebiotics is that they serve as a substrate for short chain fatty acid (SCFA) synthesis and as a consequence of SCFA production, more of the “beneficial bacterial community” is formed which further enhances the absorption of minerals (such as Ca, Zn and Fe) in the colon [12,13,50]. SCFA positively affect Fe bioavailability by releasing Fe from complexes by a lowering of the pH. The lowering of the pH enhances mineral absorption by releasing the iron from protein complexes [51]. Furthermore, one of the fermentation products, propionate, can create soluble complexes with Fe and consequently make the Fe more absorbable [51].

In addition, SCFAs can enlarge the absorption area through stimulation of the epithelial cells to proliferate. Lactobacilli and Bifidobacteria are beneficial for humans with low Fe status as bioavailability of Fe is improved as a result of the reduction in the amounts of the pathogenic becteria using dietary Fe [52].

*In-vitro* data show that more bioavailable Fe has been measured in the presence of samples containing lower levels of phytate. However, high amounts of phytate in samples were not a limiting factor for the stimulation of beneficial gut microbiata production. Generally speaking, the production of beneficial bacteria was higher in the presence of samples with higher amounts of prebiotics (wholemeal flour and bread samples) which clearly demonstrates that the consumption of wholemeal bread as opposed to white bread is not a contributing factor in the development of Fe deficiency. Similarly, the baking process (including the fermentation) does not seem to have a crucial role in microbial production as there were no discrepancies in bacterial production among the treatments of white and wholemeal flour and their corresponding baked products.

It has been shown that both bifidobacteria and lactobacilli can produce phytate degrading enzymes [53]. It seems that prebiotics influence the production of probiotics which degrades the phytate via the action of phytate degrading enzymes and in this way may positively influence Fe absorption.

Previously, we have been able to show the change of receptors of Fe in different places of the intestine in pigs and chickens in the presence of prebiotics [8,9]. However, even with those studies, it sometimes took more than six weeks to observe the changes in all Fe related parameters (i.e. the concentration of Hb). Therefore, we believe that further improvement of Fe status *in vivo* (i.e. changes in Fe related proteins and enzymes) in the presence of wheat products may be seen in a longer term study.

## Conclusion

Iron deficiency is the most prevalent nutritional deficiency worldwide. At present, there is more and more interest in improving gut health, and consequently increasing Fe absorption, by managing the colonic microbial population.

This study demonstrated that prebiotics naturally found in wheat grains/bread products significantly increased intestinal beneficial bacterial population in Fe deficient broiler chickens. The prebiotic segments of wheat enhanced the growth of colon microflora, with generally more bifidobacteria being created. This is an important finding as formed bacterial populations improve gut health and can consequently contribute to increased Fe absorption. With this short-term feeding trial we were not able to show differences in the Fe-status of broilers. Nevertheless, the bacterial analysis clearly suggests that we may have more significant differences (i.e. increased Fe bioavailability and improved Fe status) in a longer term feeding trial. Based on the results presented here, additional *in-vitro* and *in-vivo* trials are required to increase our knowledge on the nutritional and essential prebiotic properties of wheat grains and their baked products in improving Fe absorption.

## Abbreviations

Fe: Iron; Hb: Hemoglobin; Hb: Fe, hemoglobin iron; AX: Arabinoxylan; FOS: Fructooligosacharides; DH: Double haploid; SCFA: Short chain fatty acids; mRNA: Messenger ribonucleic acid; QTL: Quantitative trait loci; HPLC: High performance liquid chromatography; PCR: Polymerase chain reaction; DMT-1: Divalent metal transporter 1; DcytB: Duodenal cytochrome B; MEM: Minimum essential media.

## Competing interests

All of the authors read and approved the final version of the manuscript. None of the authors had a conflict of interest.

## Authors’ contributions

ET carried out the *in vivo* study, analyzed and interpreted data and helped to draft the manuscript, co-authored the paper. RG was responsible for *in vitro* study; reviewed the final draft of the manuscript. MK carried out preparation of bread samples and completed sugar and phytate analysis, evaluated data, wrote the manuscript and prepared the manuscript for submission to the journal. JS responsible for the design of the study, organization of micronutrient analysis, reviewed the final draft of the manuscript, co-authored the paper. All authors read and approved the final manuscript.
